# Psychological Distress and Somatization in Immigrants in Primary Health Care Practices

**DOI:** 10.3390/healthcare8040557

**Published:** 2020-12-13

**Authors:** Rosa García-Sierra, María Isabel Fernández-Cano, Josep María Manresa-Domínguez, María Feijoo-Cid, Eduard Moreno Gabriel, Antonia Arreciado Marañón, Francesc Ramos-Roure, Jordi Segura-Bernal, Pere Torán-Monserrat

**Affiliations:** 1 Research Support Unit Metropolitana Nord, Primary Care Research Institut Jordi Gol (IDIAPJGol), 08303 Barcelona, Spain; rgarciasi.mn.ics@gencat.cat (R.G.-S.); jmanresa@idiapjgol.info (J.M.M.-D.); emoreno@idiapjgol.info (E.M.G.); rourenet@gmail.com (F.R.-R.); ptoran.bnm.ics@gencat.cat (P.T.-M.); 2 Nursing Department, Faculty of Medicine, Universitat Autònoma de Barcelona, 08193 Barcelona, Spain; maria.feijoo@uab.cat (M.F.-C.); antonia.arreciado@uab.cat (A.A.M.); 3 Multidisciplinary Research Group in Health and Society GREMSAS (2017 SGR 917), 08007 Barcelona, Spain; 4 Faculty of Psychology, Education Sciences and Sport, Ramon Llull University, 08022 Barcelona, Spain; jordisb@blanquerna.url.edu; 5 Department of Medicine, Faculty of Medicine, Universitat de Girona, 17071 Girona, Spain

**Keywords:** immigrants, somatization disorders, stress, psychological, primary health care, vulnerable populations

## Abstract

The process of international migration causes a situation of vulnerability in people’s health and greater difficulty in coping with disease. Furthermore, the adversities suffered during migration can trigger reactive signs of stress and cause anxious, depressive, confusional and somatic symptoms. This article studies the relationships between psychosocial risk, psychological distress and somatization in immigrants from four communities: Maghrebis, Sub-Saharans, South Americans and South Asian. A cross-sectional study was carried out with questionnaires on 602 immigrants who were surveyed in the primary care centers of an urban area of Catalonia. The instruments used were the Demographic Psychosocial Inventory (DPSI), the Brief Symptom Inventory (BSI) and the Somatic Symptom Inventory (SSI). The average psychosocial risk obtained was 0.35, with the highest values in the Sub-Saharan community. Psychological distress showed a mean value of 0.66, with the Sub-Saharan community scoring the lowest in all dimensions except depression. The average somatization values were 1.65, with the Sub-Saharan community scoring the least. The female gender is a risk factor for somatization and psychological distress. Perceived psychosocial risk is a predictor of psychological distress, but not somatization, suggesting that the use of more adaptive coping strategies could minimize the effect of the migration process on somatizations.

## 1. Introduction

The concept of migration is defined as “The movement of persons away from their place of usual residence, either across an international border or within a State” [1]. The number of international migrants has increased in the last 50 years, and it is estimated that 272 million people live in a country different from that of their birth, triple that of 1970, with 48% of them being women [2]. In Spain, resident foreigners represent 10% of the population [3], the most recent being those from the migratory movement between 2000 and 2007 motivated by the demand for labor in the construction and domestic service sector mainly and which was later slowed by the economic crisis [4]. In 2016, there was a new rise in the number of registered resident foreigners caused by conflicts in their countries of origin and also by the reunification of families of immigrants already established in Spain. In Catalonia, population growth in recent years has been due to the immigration of the foreign population, which in 2019 represented 15% of the population [5].

The migratory process is a human experience that affects people’s health and is an emerging public health problem that needs a response. Migrating involves high levels of emotional stress that affect health and that can manifest themselves physically and psychically. It is comprised of several phases, and in each one of them, the health of immigrants is conditioned by different determinants. In the pre-migration phase, epidemiological profiles of the origin, environmental policies, and personal exposure to conflicts or human rights violations are decisive in defining the state of health. In the migratory movement phase, the duration, circumstances and conditions of the trip are the factors that most affect health. Finally, in the arrival phase, the migration policies of the destination country, access to health systems and the sensitivity of health professionals towards cultural and linguistic differences act as decisive factors on the health of migrants [6]. It should be noted that among the people who have migrated, we can find different migratory states, since some have migrated due to a well-founded fear of persecution in their country, which is why they request political asylum and can obtain refugee status and in other cases migratory causes are labor [1]. These differences may influence the determinants of the above-mentioned migratory process, and others such as loneliness, insecurity, stress suffered during the migratory trip and cultural shock upon arrival in the new country, creating a situation in which the person will have difficulties anticipating and facing the impact of disease, thereby increasing their vulnerability [7]. On the other hand, and even with having access to the health system, some groups of migrants may find it difficult to express their symptoms or understand therapeutic instructions, not only because of language barriers but also because the causal mechanisms of the disease may have different cultural constructs, especially the processes related to mental health. Furthermore, it should not be forgotten that having to move through the different healthcare levels is an added difficulty for people who come from systems of disorder [8].

All the afore-mentioned adversities can affect the person’s homeostasis and the functioning of the hypothalamic–pituitary–adrenal medullary axis, causing symptoms of psychological distress in the area of depression (sadness and crying), in the area of anxiety (irritability or insomnia), in the confusional area due to an increase in cortisol, as well as somatizations manifested by fatigue, osteoarticular discomfort or headache, among other symptoms. Somatic symptoms may appear in response to chronic stress and are also known as functional somatic symptoms (FSS), medically unexplained symptoms (MUS), bodily distress syndromes (BDS) or somatic symptom disorder (SSD). These names describe persistent physical symptoms that do not have an explanation. In relation to the manifestations associated with the migratory phenomenon, the Syndrome of Extreme Migratory Mourning was described in 1994, also called Ulysses Syndrome [9] in situations in which the chronic stress associated with the migration is extreme. Health professionals respond to somatic symptoms in a polarized way: they trivialize the symptoms due to the lack of objective evidence to explain them, due to ignorance of the syndrome or lack of cultural sensitivity, or they do not adequately diagnose the condition, classifying it as a depressive or psychotic illness, instead of a reactive picture of stress, thus elevating symptoms to the field of psychiatric pathology and adding a new stressor to the person [9,10].

Consultations for recurrent symptoms of this type are especially relevant in primary care [11], since the deterioration in the quality of life of people who suffer from them leads to 60% more use of primary care services after hours (in primary out-of-hours care) than people who do not have these diagnoses [12]. A recent systematic review argues that immigrants with somatization disorders perceive a greater need for the use of health services and greater difficulties in their daily lives than those who do not have them. However, there are differences in the prevalence of somatization disorders between different groups of immigrants, depending on culture, exposure to stress, explanatory models of the disease, coping and other individual variables [13].

Finally, gender and ethnicity are two structural social determinants of health by themselves, and their intersection may suggest synergistic effects that can be made visible when considered together. Gender is a factor associated with mental health in migrant populations. According to published literature [14,15], the probability to be affected by common mental disorder is higher among migrant women compared with men, although some studies obtained opposite results [16].

The International Organization for Migration (IOM), the WHO and the Government of Spain organized a World Consultation on the Health of Migrants in 2010 [8]. Four priorities were defined in it: monitoring the health of migrants; monitoring their equitable access to health services; developing health policies and programs that are sensitive to the migrant population; and strengthening coordination and alliances between countries. Furthermore, it should be taken into account that migrants are not a homogeneous group and that increasing knowledge about their mental health, symptoms of psychological distress and psychosomatic manifestations, while attending separately to the different communities of origin would serve as a basis to better guide the healthcare services. The objective of the study was to assess psychosocial risk, psychological distress and somatizations, as well as their main risk factors in four immigrant communities residing in Catalonia (Spain): Maghrebis, Sub-Saharans, South Americans and South Asians.

## 2. Material and Methods

### 2.1. Research Hypotheses

The research hypotheses were:

Psychosocial risk factors, psychological distress and somatizations show differences depending on the cultural community to which one belongs.

There are differences in the perception of psychosocial risk, psychological distress and somatizations, which are related to gender.

Risk factors for psychological distress will consist of female gender, older age, less time residing in the host country, less academic education and psychosocial risk before and after migration.

Risk factors for somatization will consist of female gender, older age, less time residing in the host country, less academic education and psychosocial risk before and after migration.

### 2.2. Data and Design

A cross-sectional study was designed, whose protocol has been previously published, with questionnaires administered by trained multilingual interviewers [14].

The study population were immigrants attended in the primary care centers of an urban area of Barcelona (Spain). While they were in the waiting room of the health center to be attended by their doctor or nurse, they were offered the opportunity to participate in the survey. The sampling was consecutively between November 2011 and April 2013. The inclusion criteria were that they had lived in Europe for a period of between six months and ten years, that they were from countries in North Africa, Sub-Saharan Africa, Central or South America or South Asia and that they agreed to participate voluntarily. An exclusion criterion was presenting some type of communicative difficulty, which despite the presence of a cultural mediator, did not allow for obtaining clear and understandable information.

The sample size was calculated accepting an alpha risk of 0.05, a precision of ±8% and an estimated prevalence of distress of 50%, as the case of maximum indetermination, a sample size of 151 participants is representative of each of the 4 study groups. A total of 602 people participated in the study.

### 2.3. Instruments

A form was prepared that collected sociodemographic data related to gender, marital status, educational level and length of residence in Spain. Psychosocial risk factors were collected through the Demographic Psychosocial Inventory (DPSI) [15], an 85-item inventory comprised of two scales, one referring to pre-migration status and the other to post-migration status, and with three general indexes called global index, family structure and health problems, and five dimensions that act as risk factors that promote psychological distress in immigrants. All the scales score from 0 to 1, where the highest scores represent states of poorer health or more psychosocial risk factors depending on the scale in question. The values that suggest clinical risk correspond to 0.44 for men and 0.48 for women.

Psychological distress was recorded using the Brief Symptom Inventory (BSI) [16]. In this investigation, the version adapted to Spanish [17] was used, consisting of 46 Likert-type response items from 0 to 4, according to the manifestation of symptoms in the previous 30 days, where a higher score indicates poorer mental health. The version used has six scales to encompass the primary dimensions of psycho-pathological symptoms: depression, phobic anxiety, paranoid ideation, obsession-compulsion, somatization and hostility/aggressiveness, as well as an overall score that would be a measure of psychological distress. Alpha reliabilities for the six BSI scales showed optimal indices (between 0.70 and 0.91) in the psychometric study of the Spanish version.

To detect somatic symptoms, the Somatic Symptom Inventory (SSI) was used [18], based on the DSM-III criteria for somatization disorders. It consists of 26 Likert-type response items from 0 to 4, with higher scores representing greater intensity of symptoms. According to Tamayo et al. [19], scores ≤ 52 represent minimal somatic discomfort and > 52 moderate-to-severe discomfort.

### 2.4. Ethical Considerations

The confidentiality of the data obtained was absolute and scrupulous, using no data that might identify the participants of the study. The data were filed in databases with totally anonymous information technology support and were wholly analyzed by persons other than the interviewers. The interviews were carried out in an isolated room with a relaxed atmosphere favoring the free and sincere narrative of each interviewee after first having obtained written informed consent. If necessary, cultural mediators/facilitators were required to obtain such consent. The protocol of investigation has been reviewed and approved by the Ethical and Clinical Investigation Committee of the Primary Care Research Institute IDIAP Jordi Gol (Barcelona, Spain) on 21 July 2010.

### 2.5. Data Analysis

A univariate analysis was performed calculating absolute frequency and percentage in the categorical variables and mean and standard deviation in the quantitative ones. A bivariate analysis was performed using the ANOVA test and the T test on the contrasts between variables. The predictive variables of psychosomatic symptomatology were evaluated with a multiple hierarchical regression, introducing the sociodemographic variables in the first step and the pre-migration and post-migration scales and the DPSI global indices in the second step using the PIN (0.05) and POUT (0.10) enter method. The multicollinearity of the data was analyzed using the variance inflation factor (VIF) and tolerance [20]. VIF values less than 10 in regressions and tolerance values greater than 0.10 indicate that the explanatory variables do not show multicollinearity in the chosen model. The level of significance used was *p* ≤ 0.05.

The analyses were performed with the SPSS statistical package for Windows, version 22.0 (IBM, Armonk, NY, USA).

## 3. Results

### 3.1. Univariate Analysis

A total of 602 people participated, 50% women, belonging to four communities of different origins, 20% of South American origin (Bolivia, Ecuador, Colombia), 35% of South Asian origin (Pakistan, Afghanistan, Bangladesh), 21% of North African origin (Morocco, Tunisia, Algeria) and 24% of Sub-Saharan origin (Senegal, Gambia, Mali). The composition of the sample is heterogeneous in relation to religion, educational level, marital status, employment status, knowledge of Spanish and number of children. Table 1 shows an exhaustive description of the sociodemographic characteristics of the participants according to their community of origin.

### 3.2. Bivariate Analysis

Regarding psychosocial risk factors, the global DPSI index was 0.35, showing differences between the four communities studied with people of Sub-Saharan origin perceiving greater psychosocial risk according to the a posteriori contrasts carried out (see Table 2).

Regarding the symptoms of psychological distress, Table 2 shows a description of the different scales of the BSI, as well as the overall score differentiated by communities, highlighting that Sub-Saharan immigrants have lower levels of psychological distress on the somatization, hostility, paranoid ideation and anxiety scales. On the depression scale, the lowest values were obtained by the South American community.

Psychosomatic symptoms evaluated with a single SSI score show differences between communities, with values ranging from 36 to 47, below the cut-off point indicating moderate somatic discomfort. Sub-Saharan immigrants score less than the other communities on somatization.

When comparing the pre-migration scales and the post-migration scales according to gender, differences between the two genders were observed with women having the worst results. However, the overall psychosocial risk score does not vary.

Regarding the symptoms of psychological distress and psychosomatic symptomology, women showed higher scores on all scales (see Table 3).

### 3.3. Multivariate Analysis

The regression with the psychological distress outcome variable measured with the global BSI score provides a model that explains 16.5% of the variance of psychological distress in women and 8.9% in men. The predictors in the model are religion and country of origin, the variables of post-migration scales, global DPSI index, and pre-migration scales with a positive relation, and to be married and number of years since migration with negative β (see Table 4). The predictors are different in women and men. The Table 4 shows these results disaggregate by gender.

A second hierarchical regression was performed with a psychosomatic symptomology outcome variable measured with the SSI. The chosen model explains 33.75% of the variance in psychosomatic symptomology in women and 21.9% in men. Predictor variables are age, country of origin, religion, pre-migration scales and post-migration scales with a positive relation, and number of years since migration with negative β. The global DPSI index is no longer a predictor of somatization (see Table 4). The predictors are different in women and men. The Table 5 shows these disaggregate results by gender.

## 4. Discussion

The participants in this research come mainly from nine countries (Bolivia, Ecuador, Colombia, Morocco, Tunisia, Algeria, Senegal, Gambia, Mali) grouped into four different communities: South American, Sub-Saharan, Maghrebi and Southern Asian. The cultural differences between these groups are important, although the most distinctive fact is the language: only the South American community has the same language (Spanish) as the host country.

The diversity in the origin of the participants in the sample meets two research needs agreed upon by the experts, the first to collect accurate and disaggregated data as a basis for provide evidence of the vulnerabilities of specific groups [21], the second to inquire how psychological distress may vary by sociological diversity, although no evidence shows that the epidemiology of most psychiatric disorders is changing, societal changes already underway will affect individual and population mental health [22].

The first hypothesis that was posed was that the fact of belonging to different cultural communities conditioned the perception of psychosocial risk, psychological distress and somatic symptoms. The results obtained would allow us to accept this hypothesis, since there are significant differences in the perception of psychosocial risk, as well as in all the scales of psychological distress (BSI) and in the somatization scale (SSI). In a recent systematic review [13] on somatization among immigrants, the prevalence and correlations of somatization were found to vary across the immigrant groups and mediated by cultural variation, so it was concluded that clinical management of immigrant patients should pay attention to social, cultural and linguistic differences. In addition, the a posteriori contrasts allow us to affirm a clearly differentiated behavior of Sub-Saharan immigrants compared with the other communities, since they perceive a greater psychosocial risk, although they exhibit fewer symptoms of psychological and psychosomatic distress. However, this result contrasts with those obtained in two Italian multicultural studies [23,24] in which the likelihood of somatization was significantly higher in South-Central Americans than in other ethnic groups.

The second hypothesis that was posed was the existence of differences in the perception of psychosocial risk, psychological distress and somatizations due to gender. The results partially support this hypothesis, since in all the evaluation scales, women scored significantly higher than men, showing greater pre-migration and post-migration psychosocial risk, worse psychological health and more somatization disorders. These results are in the same line as the findings of other investigations carried out with the immigrant population in which the female gender presents greater severity of somatic symptoms [24,25]. Female gender is also shown as a significant variable in anxiety, mood disorders, eating disorders and somatization, not only in immigrant communities but also in the indigenous population [11]. However, the score on the global psychosocial risk scale does not show differences between men and women. This was an unexpected result as the pre-migration and post-migration scales are part of the global scale. This difference is determined to be because the global scale adds five single items under the construct “conflict reaction”, which act to moderate psychosocial risk and as a result turn out to be more adaptive coping strategies than those used by men, thereby equalizing the global perception of psychosocial risk.

The third hypothesis supposed that the risk factors for greater psychological distress are female gender, older age, less time residing in the host country, less academic education and psychosocial risk before and after migration. The separate analysis of men and women showed differences in the predictors of psychological distress. In men, the pre-migration and post-migration conditions appear in the model; however, in women, the country of origin, religion, the global DPSI index, the years since migration and being married appear. Therefore, the results partially support the hypothesis since education and older age is not significant, the time residing in the host country and psychosocial risk is only in women as well as the cultural characteristics of origin ad religion. Furthermore, to be married appears as a predictor in women. The literature shows contradictory results in relation to the effect of educational level of people who emigrate. Having a university degree was a predictor of worse general health among refugee adults in Australia [26], while it was a protective factor in refugees, where having higher education predicted fewer medical conditions [27] and less psychological distress [28]. The pre-migration conditions in some studies had appeared as a strong predictor of depression and other psychological disorders [29]. The study by Jamil et al. (2015) [27] highlights that the pre-migration situation behaved differently in refugees and immigrants, with refugees being the main risk factor for having worse self-perceived health, whereas it did not influence the self-perceived health of the immigrant population. Regarding pre-migration conditions, Li and Anderson [30] demonstrated that the influence of the pre-migration situation on psychological symptoms was mediated by perceived discrimination, since immigrants with traumatic experiences perceive the world as a dangerous place. In other contexts, these negative beliefs about the lack of benevolence in the world lead to increased levels of vigilance, which in turn increases stress [31]. In relation to the effect of marital status, which appears with a negative relationship in women the literature also shows contradictory results, [23] found a higher risk of somatization in married people, while [32] found less somatization in married people.

The last hypothesis proposed as somatization risk factors the female gender, older age, less time residing in the destination country, lesser academic education and the psychosocial risk before and after migration. This hypothesis is partially supported, since academic education is not significant, and the years since the migration is significative only in women and the age is a predictor only in men. Further, the country of origin, the religion and the number of children are predictors in women. Evidence shows that academic education behaves ambivalently, the lower the formal education, the higher the prevalence of somatization [33], or with greater levels of education than high school, greater somatization in Russian and Hispanic immigrants [34].

The global DPSI scale is not significant for the somatization outcome variable, taking into account that the other dimensions of the perception of psychosocial risk variable, if they are significant, would be indicative that coping strategies would influence the impact that psychosocial risk may have in the onset of somatization disorders. In line with this argument, in 2008, Sachs et al. [35] explored coping strategies and stress in Tibetan refugees in India who had been exposed to traumatic situations, reporting that coping strategies acted as mediators between lived traumatic experiences and decreased somatic symptoms.

This study is the first approach to the study of somatization disorders and psychological distress of immigrants of different origins carried out in Spain. The main strength of the research is found in the composition of the sample, not only due to its high number but also due to being composed of people from 12 countries that contributed, in the year in which the research was carried out, 42% of immigrants to Catalonia [36]. The findings are applicable to immigrant populations residing in Spain and the Mediterranean area (France, Italy, Greece, Portugal) because the migratory patterns have been similar in the last fifty years.

There are some limitations in the study related to the questionnaires, the main one being the language barrier for some of the participants, since Spanish versions of the questionnaires were used, however, to minimize possible comprehension deficits, multilingual interviewers participated as cultural mediators when necessary. Second, that only the BSI questionnaire had a validated version into Spanish, the SSI and the DPSI were adapted to Spanish following a translation and reverse translation process with a subsequent evaluation of the intercultural mediators who acted as expert judges to validate the content of the questionnaires. Third, it is noted that the length of the questionnaires required an investment of time between 30 and 40 min, so we must assess the interference of fatigue in completing the questionnaires, although the fact that they were carried out by interviewers and were not self-administered would reverse part of this effect. The last limitation is related to the reasons for migration which were not collected, for we do not know if the participants are labor migrants, or family reunion reason migrant or refugees, which could be an explicative variable. In future research, these limitations could be overcome by validating the metric characteristics of the questionnaires used, by including in the study design the variable related to the motivation for the migration.

## 5. Conclusions

Despite the limitations, this research contributes to the limited literature on somatization disorders and psychological distress in different immigrant communities.

Sub-Saharan immigrants perceive a greater psychosocial risk than other cultural groups, although they show lower levels of psychological distress and lower levels of somatization.

Being a woman is a risk factor for presenting greater psychological distress and more psychosomatic symptoms. In contrast, the time that has passed since migration is a protective factor in women, but not in men. The fact that perception of psychosocial risk is a predictor of psychological distress but is not a predictor of somatic symptoms, could indicate that the coping strategies that are included in the global DPSI scale may have an influence on reducing the development of somatization disorders. Working on the coping strategies of people who have recently migrated (newcomers) might improve the presentation of psychosomatic manifestations, especially in women.

The predictors and protective factors are very different in women and men, revealing the interaction of sex with other variables, and the role that gender may play within the epidemiological studies.

## Figures and Tables

**Table 1:** Sociodemographic characteristics of the participants by community.

	South America *n* = 122	South Asia *n* = 212	Maghreb *n* = 125	Sub-Sahara *n* = 143	Total *n* = 602
Age	39.8 (11.4)	36.2 (10.0)	33.1 (10.6)	31.8 (7.6)	35.2 (10.3)
Men	58 (47.5%)	120 (56.6%)	68 (54.4%)	55 (38.5%)	301 (50.0%)
Religion					
Atheists	6 (4.9%)	0 (0.0%)	0 (0.0%)	3 (2.1%)	9 (1.5%)
Muslims	0 (0.0%)	190 (89.6%)	125 (100%)	125 (88.0%)	440 (73.2%)
Christians	116 (95.1%)	0 (0.0%)	0 (0.0%)	14 (9.9%)	130 (21.6%)
Eastern	0 (0.0%)	22 (10.4%)	0 (0.0%)	0 (0.0%)	22 (3.7%)
Level of studies					
Without studies	1 (0.9%)	28 (13.2%)	24 (19.2%)	41 (28.7%)	94 (15.6%)
Primary	15 (12.3%)	123 (58.0%)	28 (22.4%)	45 (31.5%)	211 (35.0%)
Secondary	29 (23.8%)	44 (20.8%)	26 (20.8%)	32 (22.4%)	131 (21.8%)
High school	38 (31.1%)	13 (6.1%)	19 (15.2%)	6 (4.2%)	76 (12.6%)
Job training	17 (13.9%)	0 (0.0%)	16 (12.8%)	9 (6.3%)	42 (7.0%)
University	22 (18.0%)	4 (1.9%)	12 (9.6%)	10 (7.0%)	48 (8.0%)
Civil status					
Single	34 (27.9%)	58 (27.4%)	52 (41.6%)	45 (31.5%)	189 (31.4%)
With partner	22 (18%)	0 (0.0%)	3 (2.4%)	0 (0.0%)	25 (4.2%)
Married	46 (37.7%)	144 (67.9%)	55 (44%)	90 (62.9%)	335 (55.6%)
Separate	9 (7.4%)	4 (1.9%)	1 (0.8%)	3 (2.1%)	17 (2.8%)
Divorced	10 (8.2%)	5 (2.4%)	12 (9.6%)	5 (3.5%)	32 (5.3%)
Widow/er	1 (0.8%)	1 (0.5%)	2 (1.6%)	0 (0%)	4 (0.7%)
Employment situation					
Jobless	45 (36.9%)	93 (43.9%)	53 (42.4%)	76 (53.1%)	267 (44.4%)
Street trading	0 (0.0%)	0 (0.0%)	0 (0.0%)	15 (10.5%)	15 (2.5%)
Student	4 (3.3%)	0 (0.0%)	13 (10.4%)	0 (0.0%)	17 (2.8%)
Housewife	7 (5.7%)	45 (21.2%)	14 (11.2%)	9 (6.3%)	75 (12.5%)
Active worker	63 (51.6%)	72 (34.0%)	44 (35.2%)	43 (30.1%)	222 (36.9%)
Pensioner	2 (1.6%)	2 (0.9%)	1 (0.8%)	0 (0.0%)	5 (0.8%)
Others	1 (0.8%)	0 (0.0%)	0 (0.0%)	0 (0.0%)	1 (0.2%)
Spanish knowledge					
Speak and write correctly	117 (97.5%)	17 (8.1%)	54 (43.5%)	41 (28.9%)	229 (38.4%)
Speaks and writes with difficulty	2 (1.7%)	73 (34.6%)	28 (22.6%)	43 (30.3%)	146 (24.5%)
Speak but not write	1 (0.8%)	54 (25.6%)	34 (27.4%)	43 (30.3%)	132 (22.1%)
Not speak or write it	0 (0.0%)	67 (31.8%)	8 (6.5%)	15 (10.6%)	90 (15.1%)
Years since migration	9.4 (3.3)	5.2 (3.0)	6.6 (3.0)	5.8 (2.6)	6.5 (3.4)
Number of children					
None	31 (25.4%)	86 (40.6%)	65 (52.0%)	51 (35.7%)	233 (38.7%)
1–2	68 (55.7%)	54 (25.5%)	29 (23.2%)	48 (33.6%)	199 (33.1%)
3–4	18 (14.8%)	41 (19.3%)	23 (18.4%)	35 (24.5%)	117 (19.4%)
5 or more	5 (4.1%)	31 (14.6%)	8 (6.4%)	9 (6.3%)	53 (8.8%)

**Table 2:** Mean and standard deviation of the subscales of the questionnaires and comparison by community. *N* = 602.

	M/item (SD/Item)				Total	F	Sig.
	South America	South Asia	Maghreb	Sub-Sahara			
DPSI *							
Premigration scales	0.10 (0.19)	0.16 (0.23)	0.13 (0.22)	0.15 (0.23)	0.14 (0.22)	2.30	0.077
Postmigration scales	0.43 (0.35)	0.34 (0.33)	0.35 (0.36)	0.32 (0.33)	0.36 (0.35)	2.73	0.043
Index Global DPSI	0.36 (0.09)	0.33 (0.09)	0.34 (0.07)	0.38 (0.07)	0.35 (0.09)	11.38	<0.001
BSI **							
Depression	0.78 (74)	0.89 (0.95)	1.09 (0.76)	1.09 (0.73)	0.95	4.79	0.003
Phobic anxiety	0.30 (35)	0.43 (0.71)	0.35 (0.53)	0.17 (0.24)	0.32	6.69	<0.001
Paranoid ideation	0.72 (63)	0.88 (0.97)	0.87 (0.69)	0.52 (0.42)	0.76	7.60	<0.001
Obsession–compulsion	0.65 (53)	0.82 (0.80)	0.93 (0.68)	0.67 (0.39)	0.77	5.32	0.001
Somatization	0.72 (69)	0.75 (1.00)	0.72 (0.69)	0.36 (0.35)	0.64	8.91	<0.001
Hostility/aggressivity	0.39 (58)	0.36 (0.69)	0.23 (0.45)	0.13 (0.25)	0.29	7.13	<0.001
General Severity Index	0.62 (50)	0.72 (0.80)	0.75 (0.54)	0.53 (0.29)	0.66	4.05	0.007
SSI ***	1.68 (0.63)	1.82 (1.04)	1.63 (0.52)	1.39 (0.30)	1.65 (0.75)	9.99	<0.001

Note: M/item: Mean divided by the number of items; SD/item: Standard deviation divided by the number of items; DPSI: Demographic Psychosocial Inventory; BSI: Brief Symptom Inventory; SSI: Somatic Symptom Inventory; * Score from 0 to 1; ** Score from 0 to 4; *** Score from 0 to 4.

**Table 3:** Mean and standard deviation of the subscales of the questionnaires and comparison by gender.

	M/item (SD/Item)		F	Sig.
	Female	Male		
DPSI *				
Premigration scales	0.20 (0.25)	0.07 (0.18)	54.62	<0.001
Postmigration scales	0.45 (0.33)	0.26 (0.34)	50.32	<0.001
Index Global DPSI	0.35 (0.10)	0.36 (0.08)	1.49	0.223
BSI **				
Depression	1.15 (0.90)	0.76 (0.70)	34.90	<0.001
Phobic anxiety	0.43 (0.62)	0.22 (0.41)	22.76	<0.001
Paranoid ideation	0.87 (0.82)	0.66 (0.68)	11.62	0.001
Obsession–compulsion	0.90 (0.72)	0.64 (0.55)	24.71	<0.001
Somatization	0.86 (0.90)	0.43 (0.57)	49.02	<0.001
Hostility/aggressivity	0.38 (0.63)	0.20 (0.44)	16.70	<0.001
General Severity Index	0.80 (0.68)	0.52 (0.48)	34.01	<0.001
SSI ***	1.89 (0.88)	1.42 (0.48)	64.24	<0.001

Note: M/item. mean divided by the number of items; SD/item. standard deviation divided by the number of items; DPSI: Demographic Psychosocial Inventory; BSI: Brief Symptom Inventory; SSI: Somatic Symptom Inventory; * Score from 0 to 1; ** Score from 0 to 4; *** Score from 0 to 4.

**Table 4:** Hierarchical lineal regression (enter) of psychological distress disaggregated by gender.

	*Outcome Variable BSI Women*					*Outcome Variable BSI Men*			
	Collinearity Statistics					Collinearity Statistics			
	*B*	*p*	Tolerance	FIV		*B*	*p*	Tolerance	FIV
Marital status	−0.179	0.003	0.797	1.255	Premigratin scales	0.130	0.041	0.791	1.264
Years after migration	−0.144	0.027	0.670	1.492	Postmigration scales	0.169	0.009	0.768	1.302
Religion	0.164	0.020	0.565	1.770					
Index Global DPSI	0.306	0.000	0.531	1.882					
Community	−0.274	0.000	0.546	1.832					
R^2^	0.165				R^2^	0.089			

Note: BSI: Brief Symptom Inventory; DPSI: Demographic Psychosocial Inventory.

**Table 5:** Hierarchical lineal regression (enter) of somatic symptoms disaggregated by gender.

	*Outcome Variable SSI Women*					*Outcome Variable SSI Men*			
	Collinearity Statistics					Collinearity Statistics			
	*B*	*p*	Tolerance	FIV		*B*	*p*	Tolerance	FIV
Community	−0.453	<0.001	0.539	1.854	Age	0.202	0.004	0.545	1.834
Years after migration	−0.128	0.026	0.683	1.463	Premigration scales	0.233	<0.001	0.794	1.259
Premigration scales	0.168	0.004	0.684	1.462	Postmigration scales	0.226	<0.001	0.772	1.296
Number of children	0.133	0.035	0.566	1.768					
Religion	0.222	0.001	0.554	1.804					
R^2^	0.337				R^2^	0.219			

Note: SSI: Somatic Symptom Inventory.

## Abbreviations

DPSI	Demographic Psychosocial Inventory
BSI	Brief Symptom Inventory
SSI	Somatic Symptom Inventory
FSS	Functional Somatic Symptoms
MUS	Medically Unexplained Symptoms
BDS	Bodily Distress Syndromes
SSD	Somatic Symptom Disorder
IOM	International Organization for Migration
WHO	World Health Organization
DPSI	Demographic Psychosocial Inventory
BSI	Brief Symptom Inventory
SSI	Somatic Symptom Inventory
VIF	Variance Inflation Factor
